# Dual-Tunnel Pullout Repair for the Extruded Medial Meniscus in Patients With Posterior Root Tear

**DOI:** 10.1016/j.eats.2025.103722

**Published:** 2025-07-03

**Authors:** Tsubasa Hasegawa, Yuki Okazaki, Takayuki Furumatsu, Yusuke Yokoyama, Masanori Tamura, Koki Kawada, Toshifumi Ozaki

**Affiliations:** Department of Orthopaedic Surgery, Okayama University Graduate School of Medicine, Dentistry, and Pharmaceutical Sciences, Okayama, Japan

## Abstract

Medial meniscus (MM) posterior root tear significantly disrupts knee biomechanics and often leads to rapidly progressing MM extrusion and knee joint osteoarthritis. Herein, we describe an arthroscopic repair technique—the dual-tunnel pullout repair—tailored to the treatment of MM posterior root tear with MM extrusion. We avoided the use of anchors, thereby emphasizing the cost-effectiveness and simplicity of augmentation of the meniscotibial ligament. This dual-tunnel approach enhances stability, minimizes meniscal extrusion, and decreases tension in the repaired MM, which facilitates accelerated rehabilitation. We discuss the surgical technique, advantages, limitations, and clinical implications, highlighting its utility in improving patient outcomes while addressing the challenges associated with traditional methods. This technique offers surgeons an effective and reproducible strategy for posterior root repair of the extruded MM.

Medial meniscus (MM) posterior root tear (PRT) is associated with hoop stress disruption, MM extrusion (MME), impaired load distribution, subchondral insufficiency fracture of the knee, osteoarthritis (OA), and eventual need for arthroplasty.^1^^,^^2^ Although pullout repair leads to favorable clinical outcomes,^3^, ^4^, ^5^, ^6^ controlling MME remains challenging.^7^ Techniques, such as centralization,^8^ arthroscopic belt capsulodesis,^9^ circumferential fiber augmentation,^10^ and an additional suture with two simple stitches (TSS),^11^ have been developed; however, these typically rely on costly anchors that risk failure. Nonanchor techniques often fail to control MME adequately, which is crucial for restoring native knee biomechanics.^12^ Inadequate fixation can lead to persistent extrusion, altered tibiofemoral contact mechanics, and accelerated progression of OA. Regarding another nonanchor technique, grasping the MM posterior horn directly^13^ concerns remain about overconstraint and potential alterations in knee kinematics. Although methods such as peripheral stabilization sutures reduce extrusion effectively, they may also impose excessive stabilization, highlighting the need for an optimal balance between fixation and physiological mobility.^13^

MMPRT is common in middle-aged women^12^ and is frequently associated with osteoporosis, further increasing anchor failure risk. Therefore, we present a safe, simple, and cost-effective dual-tunnel pullout (DTP) repair technique that avoids anchors and excessive MM constraint. The meniscotibial ligament (MTL) is important in addressing MME,^14^^,^^15^ and the DTP technique augments MTL from the medial to the posteromedial region.

## Surgical Technique

### Inclusion Criteria

Patients with a body mass index of <30, femorotibial angle of <180°, mild OA classified as Kellgren-Lawrence grades 0 to 2, cartilage damage limited to Outerbridge grades 0 to 2, and characteristic magnetic resonance imaging findings of MMPRT, such as cleft sign, giraffe neck sign, or ghost sign,^16^ were included.

### Standard Pullout Repair Using the TSS Technique

##### Patient Preparation

**Fig 1:**
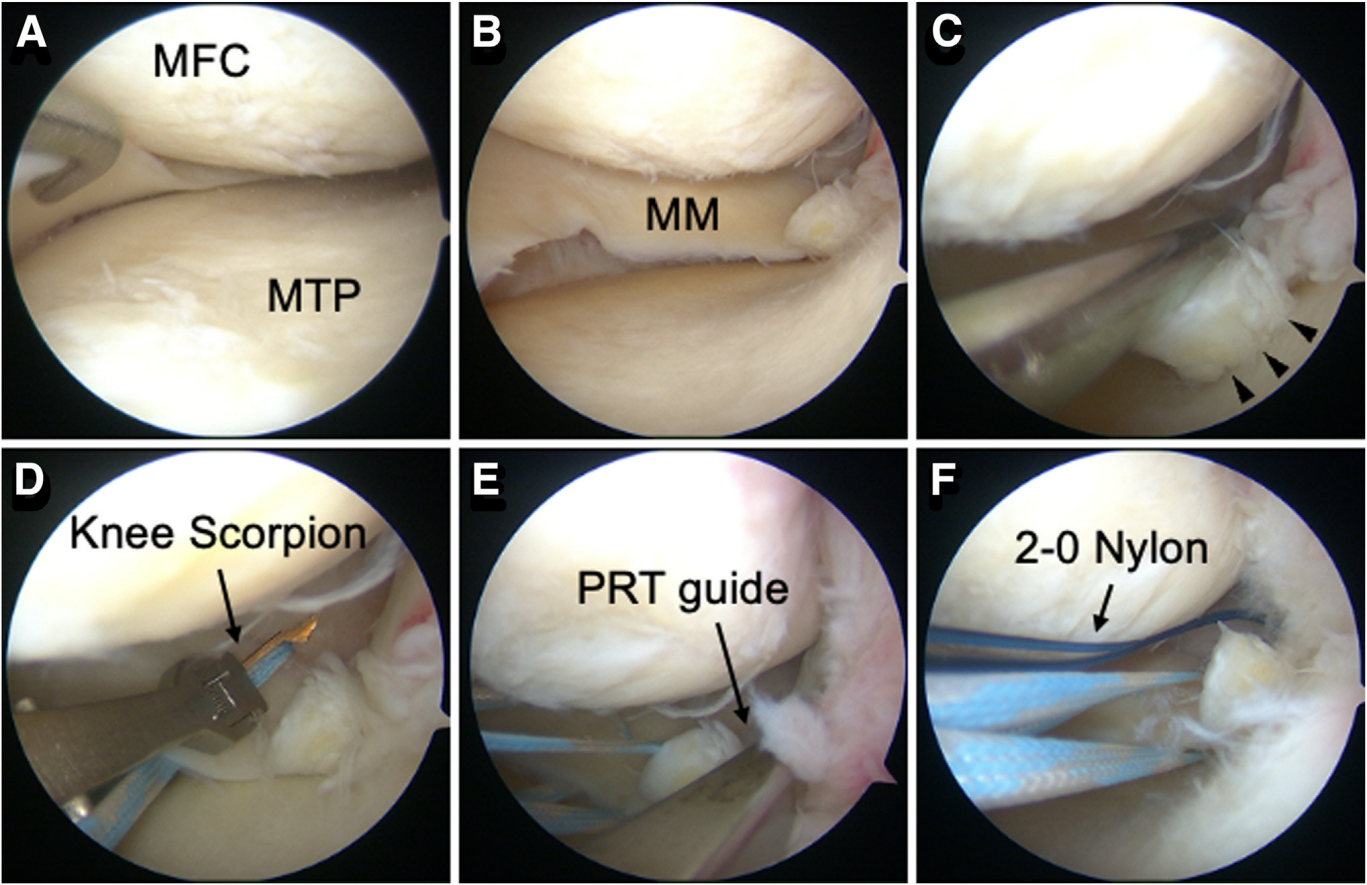
Arthroscopic findings of two simple stitches. Arthroscopic view of the left knee from the anterolateral portal. (A) Identification of the MTP edge indicating MM extrusion. (B) Creation of sufficient space for the following procedure using the pie-crusting technique. (C) Confirmation of MMPRT (LaPrade classification type 2a) using a probe (arrowhead). (D) Placement of the first suture. (E) The creation of the first bone tunnel using a PRT guide (45°) positioned at the anatomic posterior root attachment site. (F) The looped sutures are retrieved through the bone tunnel. (MFC, medial femoral condyle; MM, medial meniscus; MTP, medial tibial plateau; PRT, posterior root tear.)

A standard arthroscopic examination is performed using a 4-mm, 30° arthroscope. An MMPRT is confirmed via an anterolateral portal using a probe (Fig 1A-C).

##### Initial Space Creation

For a tight medial compartment, an outside-in pie-crusting release of the medial collateral ligament is performed using an 18-gauge needle (Fig 1B).

##### Suture Placement

The TSS technique^17^ is used. A blue/white MiniTape (Smith & Nephew, London, UK) is cut in half to create 2 tapes and passed vertically through the MM posterior horn using a Knee Scorpion suture passer (Arthrex, Naples, FL). The first inner suture is placed approximately 10 mm from the tear edge (Fig 1D), and the second outer suture is placed approximately 5 mm from it.

##### Tibial Tunnel Creation

A PRT guide (Smith & Nephew) is used to create the first tibial tunnel at the anatomic center of the posterior root attachment (Fig 1E). A 2.4-mm guide pin (Smith & Nephew) is inserted at a 45° angle and overdrilled using a 4.0-mm cannulated drill (Arthrex). Alternatively, a 2.4-mm passing pin can be used instead of a guide pin. The guide pin is removed, leaving the cannulated drill as a sleeve. A suture retriever (Smith & Nephew) is used to pass a looped suture (such as 2-0 nylon) through it (Fig 1F), facilitating the pullout procedure.

### Additional Procedure at the Posteromedial Side

##### Skin Incision

**Fig 2:**
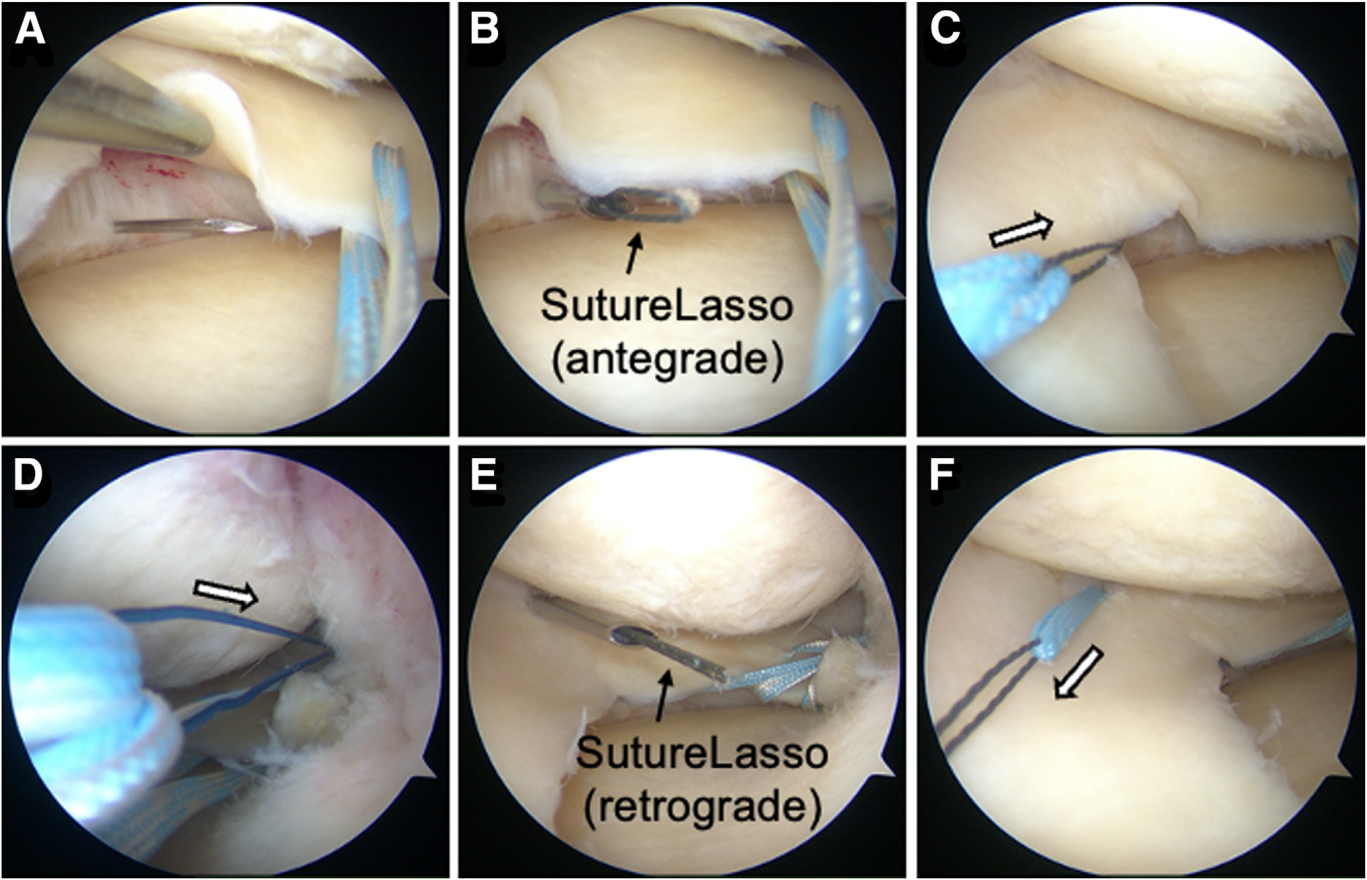
Arthroscopic findings of the additional procedure at the posteromedial portion. Arthroscopic view of the left knee from the anterolateral portal. (A) Identification of the estimated insertion site using a 22-gauge needle before making a mini-skin incision. (B) Insertion of the SutureLasso (Arthrex) beneath the posteromedial portion of the medial meniscus (MM). (C) Passage of a blue MiniTape (Smith & Nephew) beneath the MM in the antegrade direction (arrow). (D) Pulling out two blue/white MiniTapes prepared for two simple stitches, along with the intra-articular end of the blue MiniTape (2C), through the first bone tunnel (arrow). (E) Insertion of the SutureLasso above the MM. (F) Passage of the blue MiniTape over the MM in the retrograde direction (arrow). (MM, medial meniscus.)

A 22-gauge needle is inserted at the posteromedial side to confirm intra-articular positioning (Fig 2A), followed by a 3-mm skin incision.

##### Insertion of Suture Underneath the Medial Meniscus

A SutureLasso (Arthrex) is used in the antegrade direction (with the loop exiting at the needle side) to pass through a blue MiniTape underneath the MM (Fig 2 B and C). The TSS (blue/white) and intra-articular end of the MiniTape (blue) are passed through the first tibial tunnel using a left-looped suture (Fig 2D).

##### Insertion of Suture Over the Medial Meniscus

Reversing the SutureLasso (Arthrex) to retrograde (with the free end exiting at the needle side; Fig 2E) allows the blue MiniTape to be passed over the MM (Fig 2F) through the same 3-mm skin incision.

### Creation of the Second Bone Tunnel

##### Incision and Guide Setup

**Fig 3:**
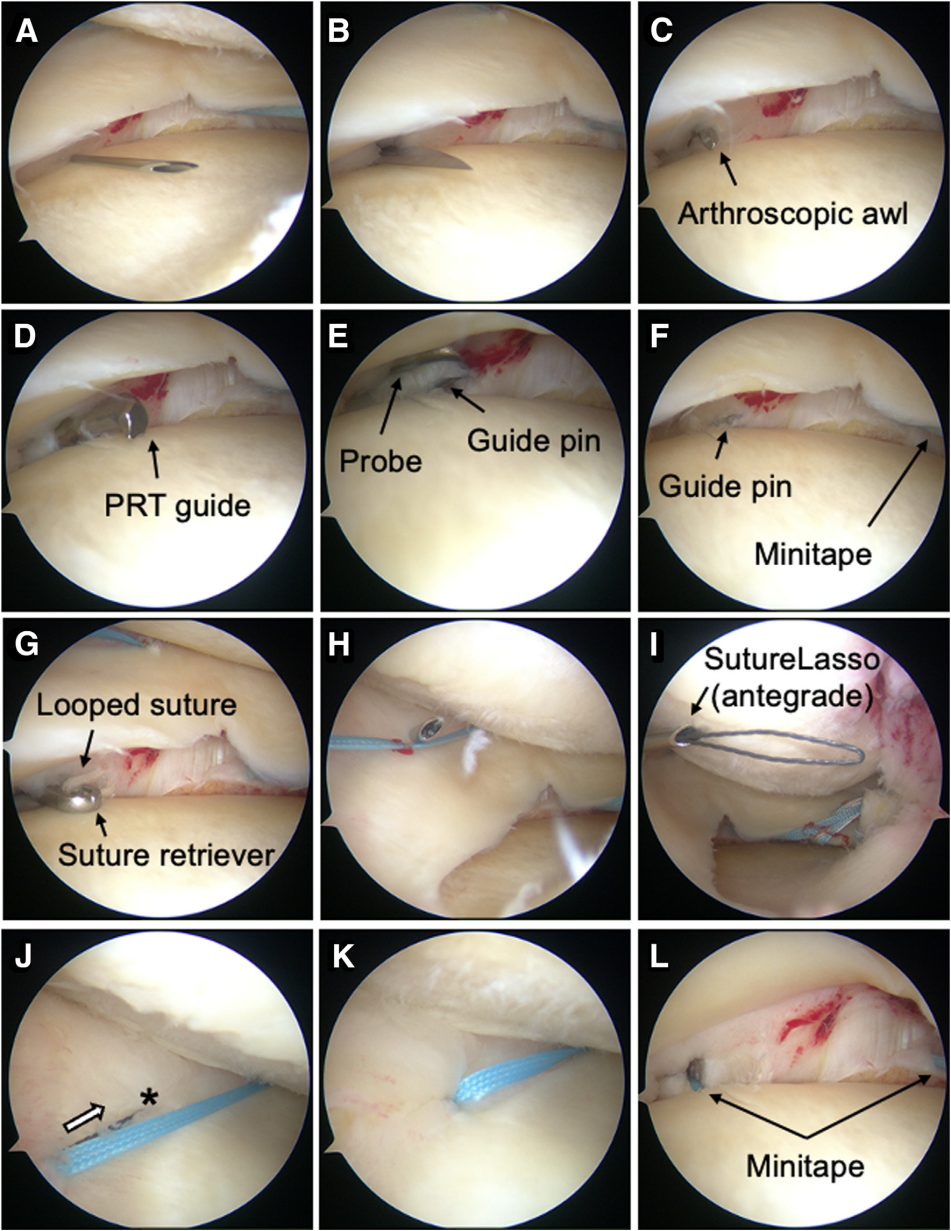
Arthroscopic findings of the additional procedure at the medial portion. Arthroscopic view of the left knee from the anterolateral portal. (A) Identification of the estimated insertion site using a 22-gauge needle before making a skin incision. (B) Creation of an accessory portal on the medial side. (C) Marking the undersurface of the medial meniscus (MM) at the edge of the medial tibial plateau using an arthroscopic awl. (D) Creation of a bone tunnel using the same PRT guide (60°), aiming at the previous bone hole. This image was taken before tunnel creation for explanatory purposes. The guide tip is hidden beneath the MM when properly positioned for tibial drilling, making it difficult to capture an image during the procedure. (E) Confirmation of the guide pin (passing pin) exiting at the edge of the medial tibial plateau. (F) Measurement of the distance between the first passing MiniTape (Smith & Nephew) and the second pin (approximately 2 cm). (G) Grasping a looped suture, which was passed using the passing pin or suture retriever (Smith & Nephew), from the medial accessory portal. (H) Insertion of the SutureLasso (Arthrex) above the MM. (I) Retrieval of the antegrade SutureLasso from the anteromedial portal. (J) Passing the MiniTape above the MM toward the asterisk-marked point (arrow). (K) MiniTape successfully passed. (L) Confirmation of the MiniTape being pulled out through the additional bone tunnel underneath the middle portion of the MM. (PRT, posterior root tear.)

A 22-gauge needle is inserted in the middle portion of the MM to confirm intra-articular positioning (Fig 3A), followed by a 10-mm skin incision (Fig 3B). When an osteophyte is present, the MTL is detached using a rasp, and the osteophyte is removed via an additional medial accessory portal and the standard anteromedial portal. An arthroscopic awl may be used to prepare the estimated site for the bone tunnel (Fig 3C).

The PRT guide is set at 60° through the medial accessory portal (Fig 3D), with the bullet tip positioned just medial to the tibial tuberosity to avoid injuring the patellar tendon. Alternatively, an anterior cruciate ligament tibial guide (Smith & Nephew) can be used instead of the PRT guide.

##### Tunnel Preparation

A 2.4-mm guide pin is inserted at the medial tibial plateau edge from a position proximal to the first bone aperture and is then enlarged using a 4.0-mm cannulated drill (Fig 3E). The pin is removed, and a suture retriever with a looped suture (such as 2-0 nylon) is inserted into the cannulated drill and retrieved through the medial accessory portal (Fig 3G). Alternatively, a 2.4-mm passing pin (Smith & Nephew) can be used without enlarging the tibial aperture.

##### Preparation for the MiniTape Pullout

The SutureLasso is inserted in the antegrade direction through the 10-mm medial accessory portal over the MM (Fig 3 H and I). The blue MiniTape is then passed over the middle portion of the MM (Fig 3 J and K) and pulled out through the second bone tunnel using a left-looped suture (Fig 3L).

##### Suture Tightening and Fixation

**Fig 4:**
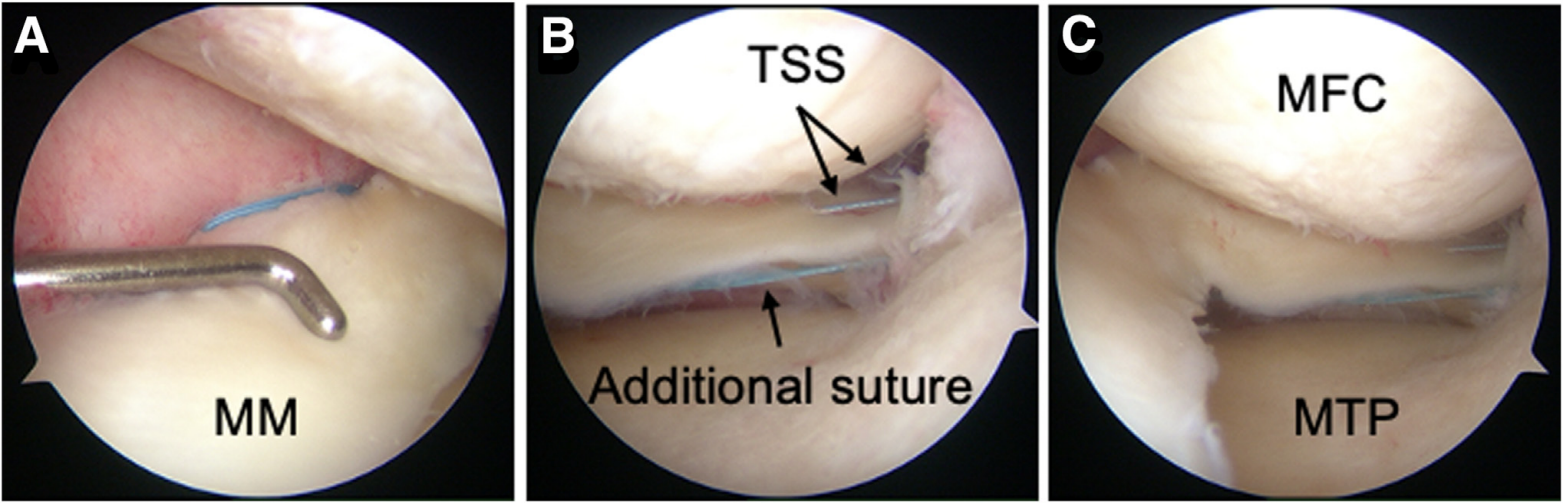
Arthroscopic findings after fixation. Arthroscopic view of the left knee from the anterolateral portal. (A) Confirmation that the blue MiniTape (Smith & Nephew) is positioned above the MM without penetrating it. (B) Confirmation of the repaired MM posterior root. (C) Confirmation that the MTP is sufficiently covered by the MM. (MFC, medial femoral condyle; MM, medial meniscus; MTP, medial tibial plateau; TSS, two simple stitches.)

The blue MiniTape is tightened to the mid-posteromedial portion of the MM with maximal manual tension (70-80 N) near the second aperture to avoid impingement with the interference screw at the first aperture (Fig 4A).

### Final Fixation

Maintaining 30° of knee flexion, pullout sutures are secured using a 5.0-mm or 6.0-mm bioabsorbable screw under initial tension (20 N) (Fig 4 B and C). Typically, a 6.0-mm screw is recommended due to the presence of osteoporotic bone.

**Fig 5:**
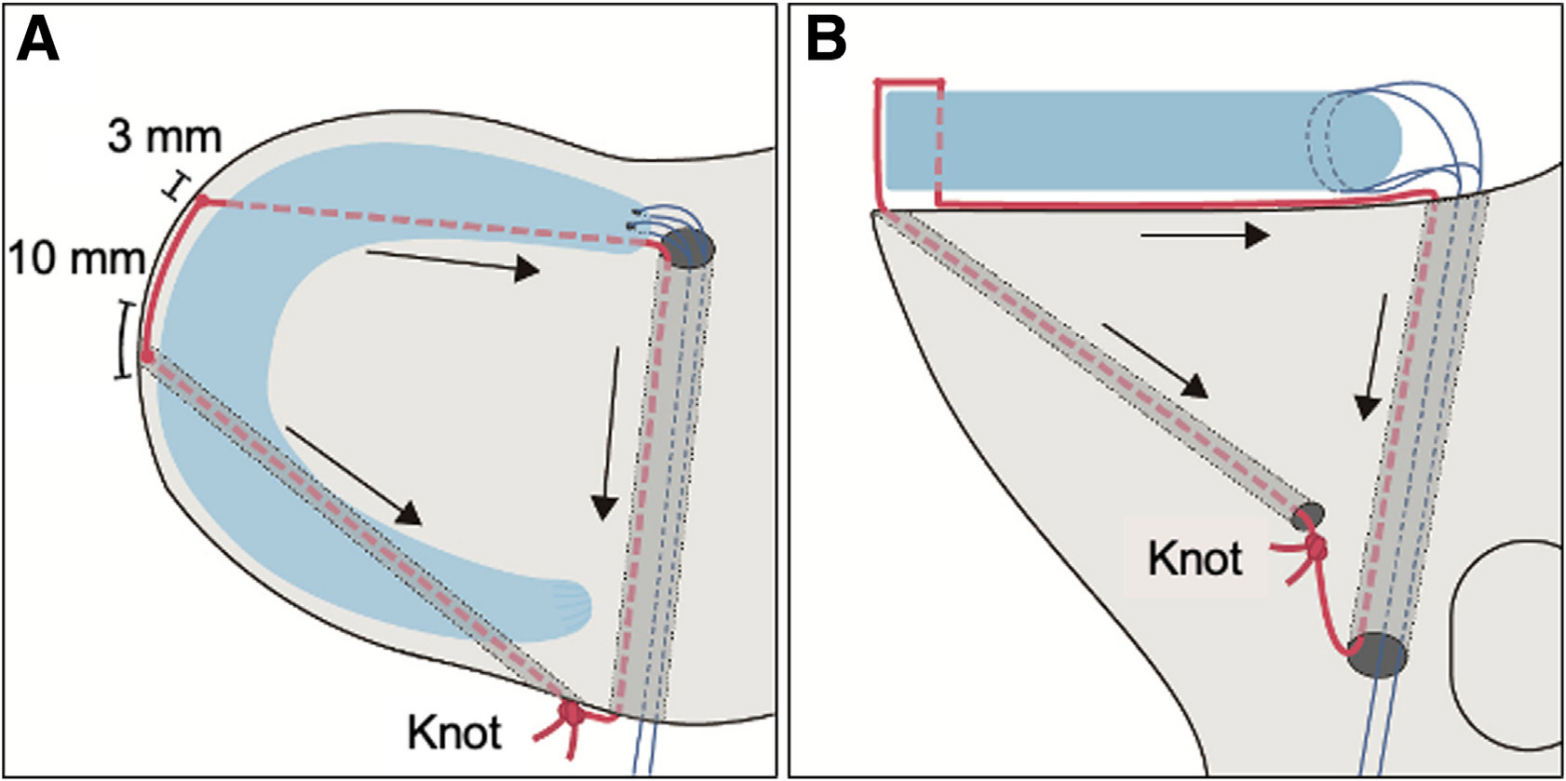
Schematic diagrams. (A) Superior view. (B) Anterior view. Blue lines show tapes for two simple stitches, and red line indicates an additional tape.

A schematic diagram is presented in Figure 5, and a detailed video demonstration is available (Video 1). The advantages, disadvantages, and key technical tips/pitfalls of this technique are summarized in Tables 1 and 2.

**Table 1:** Advantages and Disadvantages

Advantages	Disadvantages
Cost-effective, no anchors needed	Requires careful suture management
Targets extrusion in critical areas (medial and posteromedial portion)	May require assistance
Simplifies procedure with small incisions	Need additional material costs (tape)
Supports faster rehabilitation	Risk of overcontrolling movement
Rarely removes osteophyte and releases the meniscotibial ligament, as the indication for pullout repair is mild osteoarthritis	
This technique can be adopted for MM extrusion without MM posterior root tear. The bone tunnel can be created under the MM mid-portion, similar to this report, and an additional MM posteromedial portion.	

MM, medial meniscus.

**Table 2:** Tips and Pitfalls

✓ To obtain sufficient medial joint space, perform pie-crusting; however, improper execution may injure the MCL.
✓ Keep the long tape for MTL augmentation to fasten sufficiently.
✓ The smaller 3-mm skin incision should be made securely from the posterior side to ensure that the tape is hidden beneath the meniscus. ✓ For the second bone tunnel: 1. Prepare the estimated site using an arthroscopic awl, which also helps prevent the guide tip from slipping. 2. To protect the patellar tendon during a guide pin insertion and drilling, position the bullet tip just medial to the tibial tubercle.
✓ Since the SutureLasso is inserted into the joint multiple times, utmost care should be taken to avoid damaging the MM and cartilage of the MFC and MTP. ✓ When performing suture relay, ensure that the suture and the lasso wire exit through the same portal to prevent them from getting caught in the fat pad or synovium around the portal. ✓ If an osteophyte is present or MME is severe, thoroughly detach the MTL and remove the osteophyte to ensure proper reduction of the MM within the joint.

MCL, medial collateral ligament; MFC, medial femoral condyle; MM, medial meniscus; MME, medial meniscus extrusion; MTP, medial tibial plateau; MTL, meniscotibial ligament.

### Postoperative Rehabilitation

On the first postoperative day, partial weightbearing (PWB) of approximately 25 kg is permitted, with the knee range of motion limited to 0° to 30°. At 1 week, PWB is increased to 50 kg, and knee flexion is allowed up to 60°. At 2 weeks, PWB is further increased to 75 kg, with knee flexion limited to 90°. At 3 weeks, PWB is increased to 100 kg. At 4 weeks, knee range of motion is increased to 0° to 120°.

## Discussion

The DTP repair technique offers significant advancements in addressing the MME. Utilizing the dual-tunnel approach ensures greater stability and control over suture translation, which is critical for restoring meniscal and meniscotibial function and improving postoperative recovery.

This technique offers several advantages: it is cost-effective because it avoids anchors; it targets extrusion control by focusing on the most extruded portion of the MM during knee flexion, particularly the posteromedial portion^11^^,^^18^; it is simpler and less invasive, requiring fewer and smaller incisions; the tunnel for the MMPRT also facilitates MTL augmentation; and it effectively suppresses the MME, distributes stress to the artificial tape used for augmentation, decreases stress on the posterior root, and allows for accelerated rehabilitation. Adequate control of the MME with this technique may potentially extend its applicability to patients with varus alignment that would traditionally require osteotomies or unicompartmental knee arthroplasty, such as a femorotibial angle of ≧180°, hip-knee-ankle angle of ≧4 or 5°,^19^^,^^20^ or mechanical axis of ≦30%,^21^ thereby broadening the surgical indications.

An additional advantage of this technique is its ability to apply a higher tension of 70 to 80 N compared to 30 to 40 N, which is known to improve the MME.^22^ Increasing the applied force beyond this threshold may further reduce extrusion and better stabilize the posterior root, mitigating the risks of joint space narrowing and OA progression. Recent biomechanical studies have confirmed that supplemental centralization—achieved through either triple-anchor fixation^23^ or a dual-tunnel construct^24^—significantly decreases the MME and medial compartment contact pressures across 30° to 60° of knee flexion. These findings support the rationale for using higher tension and a dual-tunnel configuration in clinical practice. Moreover, reduced MME may decrease suture translation for the MMPRT, minimizing the load on the posterior root and contributing to the stability and durability of the repair.

However, certain challenges and limitations remain. The procedure involves complex suture management, requiring careful attention to avoid entangling multiple sutures. It also depends on adequate assistance and a well-coordinated surgical team. The cost of additional materials, such as tapes and the SutureLasso, makes it slightly more expensive compared to the standard TSS technique. Furthermore, there is a risk of overcontrolling the MTL, which could restrict physiological MM movement and compromise natural joint kinematics. Finally, long-term success depends on careful postoperative monitoring of the medial joint space and MM posterior root healing.

In conclusion, DTP repair represents a significant step forward in the management of MMPRT with MME, and its advantages include simplicity, cost-effectiveness, targeted extrusion control, and minimal invasiveness. Despite its limitations, it remains a promising alternative to anchor-based methods, offering reliable stabilization and enhanced recovery outcomes. Further studies are required to validate its efficacy across diverse patient populations and explore potential refinements to address the current limitations.

## Disclosures

All authors (T.H., Y.O., T.F., Y.Y., M.T., K.K., T.O.) declare that they have no known competing financial interests or personal relationships that could have appeared to influence the work reported in this paper.

## Supplementary Data

Video 1 Dual-tunnel pullout repair surgical technique for the extruded medial meniscus (MM) posterior root tear (PRT). The patient’s left knee is visualized using arthroscopy from the anterolateral portal. This narrated video demonstrates diagnostic arthroscopy confirming MM PRT with associated MM extrusion. Pie-crusting release of the medial collateral ligament is performed to improve the medial joint space, followed by the suture placement using a 2-simple-stitch technique with MiniTape (Smith & Nephew) in the MM posterior horn/root. The first tibial tunnel is created at the anatomic footprint of the MM posterior root, through which the sutures are retrieved. Additional posteromedial access is established to allow 1 suture to pass beneath and the other over the meniscus. A second tibial tunnel is then created at the medial edge of the medial tibial plateau. The MiniTape is passed over the middle portion of the MM and retrieved through this second tunnel. Finally, the sutures are tied.
